# NGF-TrkA Axis Enhances PDGF-C-Mediated Angiogenesis in Osteosarcoma via miR-29b-3p Suppression: A Potential Therapeutic Strategy Using Larotrectinib

**DOI:** 10.3390/life15010099

**Published:** 2025-01-15

**Authors:** Sheng-Mou Hou, Ching-Yuan Cheng, Wei-Li Chen, En-Ming Chang, Chih-Yang Lin

**Affiliations:** 1 Department of Research, Taiwan Blood Services Foundation, Taipei 111, Taiwan; m009660@ms.skh.org.tw; 2 The Director’s Office, Shin Kong Wu Ho-Su Memorial Hospital, Taipei 111, Taiwan; 3 Division of Chest Medicine, Shin Kong Wu Ho-Su Memorial Hospital, Taipei 111, Taiwan; m009437@ms.skh.org.tw; 4 Graduate Institute of Anatomy and Cell Biology, College of Medicine, National Taiwan University, Taipei 100, Taiwan; willy10518@gmail.com; 5 Department of Respiratory Care, Shin Kong Wu Ho-Su Memorial Hospital, Taipei 111, Taiwan; t008856@ms.skh.org.tw; 6 Translational Medicine Center, Shin Kong Wu Ho-Su Memorial Hospital, Taipei 111, Taiwan

**Keywords:** osteosarcoma, NGF, larotrectinib, angiogenesis, miR-29b-3p

## Abstract

Angiogenesis plays a critical role in osteosarcoma (OS) growth and metastasis. While nerve growth factor (NGF) is implicated in cancer progression, its role in OS angiogenesis remains unclear. This study explored NGF’s effects on angiogenesis and the underlying molecular mechanisms. Analysis of GEO (GSE16088) data identified five angiogenesis markers significantly upregulated in OS tissues. In vitro experiments demonstrated that NGF enhanced HUVEC tube formation by upregulating platelet-derived growth factor C (PDGF-C) expression and suppressing microRNA-29b-3p (miR-29b-3p). The results of tube formation assays confirmed that NGF stimulation significantly increased the angiogenic capacity of MG63/NGF cells compared to MG63 cells. Furthermore, larotrectinib, a TrkA inhibitor, effectively reduced the migration and invasion abilities of MG63/NGF cells in a dose-dependent manner. These findings suggest that the NGF-TrkA axis promotes PDGF-C-mediated angiogenesis by inhibiting miR-29b-3p signaling. Larotrectinib could serve as a potential therapeutic agent targeting NGF-mediated angiogenesis in OS, offering a promising avenue for treatment.

## 1. Introduction

Osteosarcoma (OS) is the most common primary malignant bone tumor, predominantly affecting children and adolescents [1,2]. Originating from mesenchymal cells, OS is highly aggressive and prone to early metastasis, resulting in poor prognosis [3,4]. The most common tumor sites include the distal femur, proximal tibia, and proximal humerus, with a higher incidence in males (male-to-female ratio of 1.5–2:1) [5]. Advances in surgery and neoadjuvant chemotherapy have improved the five-year survival rate for non-metastatic OS to 60–70%. However, for patients with metastasis, which is present in 20–30% of cases at diagnosis, survival drops below 30% [6,7]. OS frequently metastasizes to the lungs, and its resistance to conventional chemotherapies exacerbates treatment challenges [8]. Although chemotherapy with agents such as methotrexate, doxorubicin, and cisplatin remains the cornerstone of treatment, 20–40% of patients experience recurrence or distant metastases [9]. These limitations underscore the urgent need for novel biomarkers and therapeutic targets to improve outcomes. Current research focuses on understanding the molecular mechanisms underlying OS progression to identify new pharmacological interventions that can enhance existing treatments and improve survival rates, particularly for high-risk and metastatic patients.

OS is a highly vascularized and aggressive cancer characterized by genomic complexity, extensive heterogeneity, and metastatic dissemination primarily through blood vessels [10]. Angiogenesis plays a critical role in OS progression by enabling tumor growth, proliferation, and metastasis through oxygen and nutrient delivery [11,12]. Tumor cells in the microenvironment secrete pro-angiogenic factors, such as vascular endothelial growth factor (VEGF), which activate endothelial cells to produce proteolytic enzymes. This facilitates extracellular matrix degradation, endothelial cell proliferation, migration, and the formation of new blood vessels [13]. VEGF overexpression has been linked to increased microvascular density, metastasis, and poor survival outcomes in OS [10,11]. However, some studies report conflicting findings regarding its prognostic value. Anti-angiogenic therapies targeting VEGF-related pathways, such as bevacizumab and sorafenib, have shown promise in preclinical and clinical studies, suggesting their potential in treating metastatic OS [13]. Despite these advancements, further research is needed to identify precise targets and optimize risk stratification for anti-angiogenic therapies. Understanding the role of angiogenesis in OS progression provides valuable insights into therapeutic strategies for this rare but aggressive cancer.

MicroRNAs (miRNAs) are small, non-coding RNAs, approximately 22 nucleotides in length, that regulate gene expression at the post-transcriptional level [14,15]. By binding to complementary sequences in the 3′-untranslated regions (UTRs) of target mRNAs, miRNAs can promote mRNA degradation, inhibit translation, or, in some cases, activate it [14,16]. These regulatory molecules are involved in various biological processes, including cell proliferation, differentiation, apoptosis, migration, and angiogenesis, playing critical roles in both normal physiology and pathological conditions such as cancer, autoimmune diseases, fibrosis, and neovascularization [17,18]. Dysregulated miRNA expression is strongly associated with tumor progression, metastasis, and therapeutic resistance. For example, miR-18b-5p and miR-331-3p exhibit tumor-suppressive functions in OS [15,16], while the miR-29 family (miR-29a, miR-29b, miR-29c) regulates endothelial cell function and angiogenesis [17]. MiR-29b suppresses tumorigenesis and angiogenesis in breast cancer by targeting Akt, while miR-29c modulates HUVEC proliferation and tube formation by inhibiting insulin-like growth factor-1 signaling [19,20]. Given their therapeutic potential, miRNAs have emerged as biomarkers and drug targets, with several miRNA-based therapies, such as miravirsen (a miR-122 inhibitor), undergoing clinical trials for cancer and other diseases [21]. This study aims to determine whether miRNAs affect OS angiogenesis and elucidate the underlying mechanisms.

The nerve growth factor (NGF) and its receptor, tropomyosin receptor kinase A (TrkA), play critical roles in tumor progression, including angiogenesis, metastasis, and cancer-induced bone pain [22]. TrkA, a high-affinity receptor for NGF, regulates various downstream signaling pathways, such as RAS/MAPK, PI3K/AKT, and PLCγ, which promote cell proliferation, differentiation, survival, and tumor vascularization [23,24]. NGF is highly expressed in the tumor microenvironment and facilitates cancer progression by promoting the growth of sensory and sympathetic nerves [25]. The NGF/TrkA axis has been implicated in the progression and metastasis of multiple cancers, including breast, lung, colon, pancreatic, and prostate cancers [24,25]. In OS, NGF-TrkA signaling is suspected to enhance angiogenesis by influencing endothelial and tumor cells, although the molecular mechanisms remain unclear. Larotrectinib, a highly selective inhibitor of the TRK family, including TrkA, has shown promising efficacy in TRK fusion-positive cancers [5,26]. Its potential to inhibit NGF/TrkA-mediated angiogenesis in OS offers a novel therapeutic strategy. The aim of this study is to investigate the role of the NGF-TrkA axis in OS angiogenesis and to elucidate the molecular mechanisms driving this process, focusing on its interaction with platelet-derived growth factor C (PDGF-C) and miR-29b-3p. Understanding these pathways could provide a basis for developing novel therapeutic strategies targeting NGF-mediated angiogenesis in OS. By targeting this pathway, larotrectinib may suppress tumor-induced angiogenesis and metastasis, providing a new avenue for OS treatment. Further research into NGF/TrkA signaling and the effects of larotrectinib could advance therapeutic options for this aggressive cancer.

## 2. Materials and Methods

### 2.1. Materials

NGF recombinant protein was obtained from PeproTech (Rocky Hill, NJ, USA). All cell culture mediums and supplements were obtained from Invitrogen (Carlsbad, CA, USA). Larotrectinib (Catalog No: HY-12866) was purchased from MedChemExpress (Monmouth, NJ, USA).

### 2.2. Analysis of mRNA Expression Profiles from the Gene Expression Omnibus (GEO)

Gene expression data (GSE16088) were obtained from the GEO database, which includes comprehensive gene expression profiles from 14 human osteosarcoma tumor tissue samples and three normal tissues (kidney, liver, and lymph node) [5,27].

### 2.3. Cell Cultures

Human MG63 osteosarcoma cell lines were procured from the American Type Culture Collection (ATCC) (Manassas, VA, USA). All osteosarcoma cells were cultured in DMEM medium supplemented with 10% FBS and antibiotics. The cells were maintained in a humidified incubator at 37 °C with 5% CO_2_ [28].

Human umbilical vein endothelial cells (HUVECs; HBCRC No. H-UV001) were procured from the Bioresource Collection and Research Center (BCRC) in Hsinchu, Taiwan. These cells were cultured in a human large vessel endothelial cell basal medium with streptomycin (100 μg/mL), penicillin (100 U/mL), and 10% FBS and maintained at 37 °C in a 5% CO_2_ environment [28].

### 2.4. MicroRNA Database Searches

To predict miRNAs that could potentially target the PDGF-C gene, we employed the miRNA database miRWalk (http://mirwalk.umm.uni-heidelberg.de/, accessed on 15 October 2024). Incorporating miRDB, TargetScan, and miRTarBase databases and setting the filter to a minimum threshold of 0.85, we identified miR-29b-3p as a candidate with potential binding affinity to the PDGF-C gene. The sequences of primers are listed in Table 1.

### 2.5. Quantitative Real-Time Polymerase Chain Reaction (qRT-PCR)

Total RNA was extracted from MG63 osteosarcoma cells using the TRIzol kit (MDBio Inc., Taipei, Taiwan). cDNA synthesis was performed using the Invitrogen reverse transcription kit (Carlsbad, CA, USA). Real-time PCR analysis was conducted with 2 μL of cDNA template, sequence-specific primers, and SYBR Green PCR Master Mix (Thermo Scientific, Waltham, MA, USA) in a total reaction volume of 20 μL. Relative gene expression was calculated using the 2^−ΔΔCt^ method, with normalization to glyceraldehyde 3-phosphate dehydrogenase (GAPDH) [29]. Primer sequences were designed using a PrimerBank and listed in Table 1.

### 2.6. Cell Transfection

Silencing PDGF-C siRNA (sc-39707) and control siRNA (sc-37007) were purchased from Santa Cruz Biotechnology (Santa Cruz, CA, USA). Control mimic, miR-29b-3p mimic, and Lipofectamine 2000 were obtained from Invitrogen (Carlsbad, CA, USA). Transient transfection of siRNA or miRNA mimic was performed using Lipofectamine 2000 in accordance with the manufacturer’s protocol. Osteosarcoma cells were seeded in a 6-well plate (5 × 10^5^ cells per well) and cultured for 16 h until reaching 80% confluence. One hour prior to transfection, the culture medium was replaced with serum- and antibiotic-free medium. Cells were incubated with transfection mixtures containing 100 nM silencing siRNA or control siRNA for 24 h.

To establish an osteosarcoma cell line overexpressing NGF, MG63 cells were transfected with the pcDNA3.1/NGF vector (MDBio Inc.) using Lipofectamine 2000. At 48 h post-transfection, 200 μg/mL G418 (Geneticin, Life Technologies, Carlsbad, CA, USA) was added for selection and administered twice weekly over three weeks. Once the MG63/NGF cell line was established, gene expression was assessed using qPCR to confirm expression levels.

### 2.7. Harvesting OS Conditioned Medium (CM)

Human MG63 cells were plated in 6-well plates at 2 × 10^5^ cells/well density in a culture medium until 80% confluence. Cells were transfected with genetic siRNA or miRNA mimic, followed by NGF treatment. Subsequently, the resulting CM was collected and centrifuged to remove the pellets. The final CM was stored at −80 °C for later use.

### 2.8. HUVEC Cells Tube Formation and Quantification

Pre-coated 120ul Matrigel to 48 plates well for 30 min. HUVECs were seeded at a density of 3 × 10^5^ cells in a medium consisting of a 50/50 mixture of EGM™ 2MV and conditioned medium (CM) from MG63 cells. The cell mixture was then plated onto Matrigel-coated plates. After a 6-h incubation period, tube formation by HUVECs was visually documented through photography, and the total number of tube branches was manually quantified.

### 2.9. Statistical Analysis

Data are presented as mean ± standard deviation (SD). Statistical analysis was performed using the two-tailed Student’s *t*-test to evaluate differences between groups, with significance set at *p* < 0.05.

## 3. Results

### 3.1. NGF Induces the Expression of PDGF-C to Promote Angiogenesis

**Figure 1:**
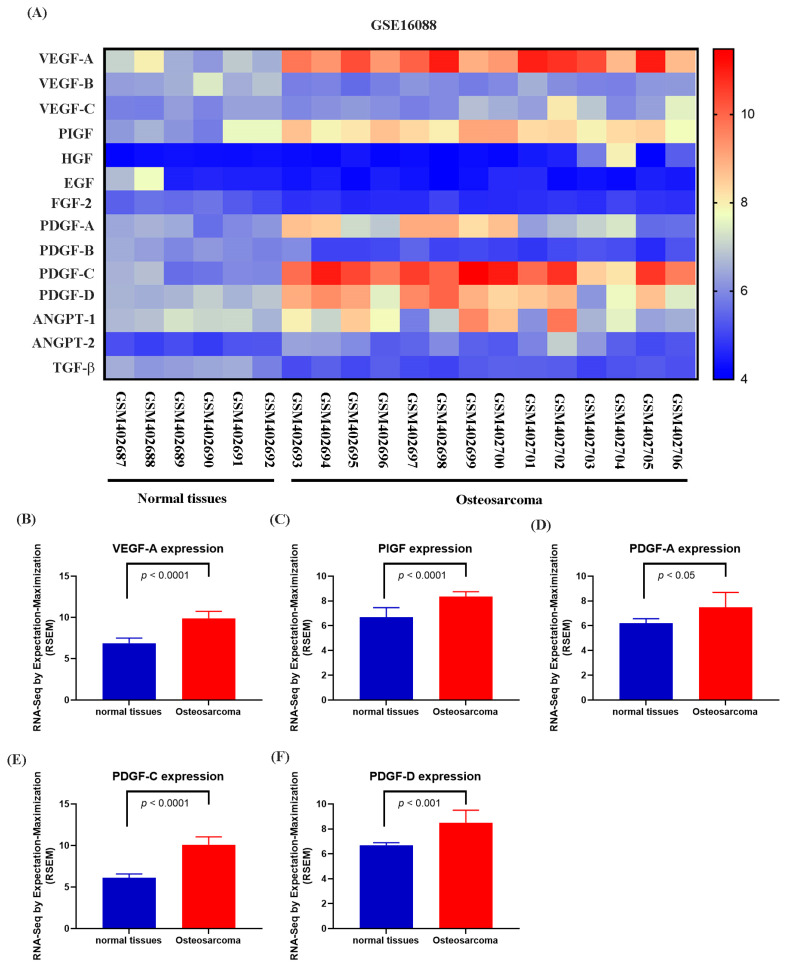
Differential expression of angiogenesis-related genes in osteosarcoma and normal tissues (GSE16088 dataset). (**A**) Heatmap showing the expression levels of angiogenesis-related genes, including the VEGF family (VEGF-A, VEGF-B, VEGF-C), PlGF, HGF, EGF, FGF-2, PDGF family (PDGF-A, PDGF-B, PDGF-C, PDGF-D), ANGPT family (ANGPT-1, ANGPT-2), and TGF-β, in normal and osteosarcoma tissues from the GSE16088 dataset. Expression values are represented on a color scale, with red indicating higher expression and blue indicating lower expression. (**B**–**F**) Differential expression analysis of selected genes between normal and osteosarcoma tissues. Quantitative analysis shows significant upregulation of VEGF-A (**B**), PlGF (**C**), PDGF-A (**D**), PDGF-C (**E**), and PDGF-D (**F**) in osteosarcoma tissues compared to normal tissues. Data are presented as mean ± SEM based on RNA-Seq expression values normalized to RPKM.

**Figure 2:**
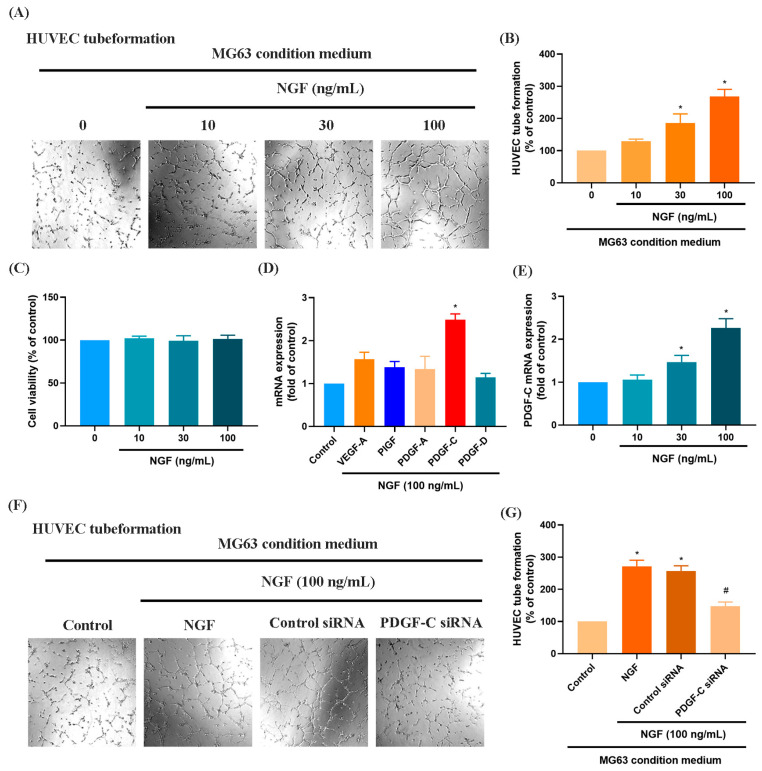
NGF promotes angiogenesis in MG63 cells through PDGF-C expression. (**A**,**B**) HUVEC tube formation assays showing the growth of capillary-like structures following 6-h incubation in conditioned medium collected from MG63 cells treated with NGF at various concentrations (0–100 ng/mL) for 24 h. Images were captured at 4× magnification. (**C**) MG63 cells were treated with different concentrations of NGF (10, 30, 100 ng/mL) for 24 h, and cell viability was measured using the MTT assay. (**D**) MG63 cells were incubated with NGF for 24 h before determining 5 potential angiogenesis candidates by qPCR. (**E**) MG63 cells were incubated with varying concentrations of NGF (30, 50, or 100 ng/mL) for 24 h, and the levels of PDGF-C expression were examined by qPCR assays. (**F**,**G**) HUVEC tube formation assays showing the growth of capillary-like structures following 6-h incubation in conditioned medium collected after MG63 cells were transfected with PDGF-C siRNAs for 24 h, then stimulated with NGF for an additional 24 h. Images were captured at 4× magnification. All experiments were repeated at least three times. * *p* < 0.05 compared with the control group; # *p* < 0.05 compared with the NGF-treated group.

Numerous studies have demonstrated that elevated VEGF and PDGF family expression levels are strongly associated with poor prognosis following bone cancer metastasis [30,31]. The role of PDGF-C in promoting osteosarcoma (OS) progression, angiogenesis, and lung metastasis has been well established [32]. RNA sequencing data from the GSE16088 dataset revealed significant upregulation of angiogenesis-related genes, particularly VEGF and PDGF family members, in OS tissues compared with three normal tissues, supporting their critical roles in OS angiogenesis (Figure 1A–F). Conditioned medium (CM) from NGF-treated OS cells promoted HUVEC tube formation in a dose-dependent manner (Figure 2A,B). NGF stimulation of MG63 cells revealed no significant effect on cell proliferation (Figure 2C). However, NGF significantly increased PDGF-C expression in MG63 cells without notable effects on other angiogenic factors (Figure 2D). Furthermore, NGF enhanced PDGF-C mRNA expression in a dose-dependent manner (Figure 2E). Transfection with PDGF-C siRNA significantly reduced HUVEC tube formation (Figure 2F,G). As shown, the dose-dependent effects of NGF observed in Figure 2B,E suggest that NGF plays a critical role in modulating angiogenesis by regulating PDGF-C expression. Increasing concentrations of NGF significantly enhance the angiogenic capacity of HUVECs and the expression of PDGF-C in MG63 cells, supporting a direct relationship between NGF levels and pro-angiogenic signaling. These results indicate that NGF promotes angiogenesis in OS cells by upregulating PDGF-C expression.

### 3.2. The miR-29b-3p/PGDF-C Axis Regulates NGF-Enhanced Angiogenesis in MG63 Cells

**Figure 3:**
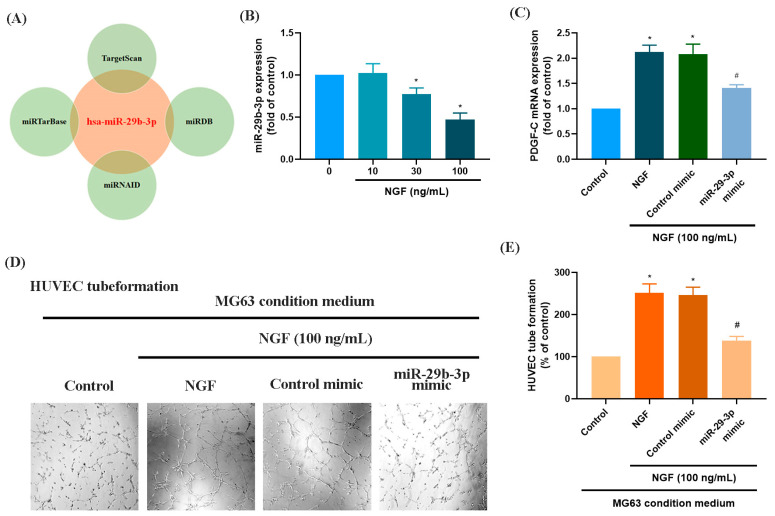
NGF increases PDGF-C expression and HUVEC tube formation in MG63 cells by downregulating miR-29b-3p. (**A**) An analysis of four miRNA prediction databases identified miR-29b-3p as a candidate binding to the 3′-UTR of PDGF-C. (**B**) MG63 cells were treated with different concentrations of NGF (30, 50, 100 ng/mL) for 24 h, and miRNA expression levels were examined by qPCR. (**C**–**E**) MG63 cells were transfected with miR-29b-3p mimic or control mimic for 24 h, then stimulated with NGF for another 24 h. PDGF-C expression levels and angiogenesis were evaluated using qPCR and HUVEC tube formation assays. Images were captured at 4× magnification. All experiments were repeated at least three times. * *p* < 0.05 compared with the control group; # *p* < 0.05 compared with the NGF-treated group.

MicroRNAs are recognized as key regulators of cancer cell progression, metastasis, and proliferation, and they hold potential as prognostic markers [33,34]. Using four online databases (miRTarBase, TargetScan, miRNAID, and miRDB), we identified a promising candidate miRNA, miR-29b-3p, which targets the 3′-UTR of PDGF-C mRNA (Figure 3A). Exposure of MG63 cells to increasing concentrations of NGF (30, 50, or 100 ng/mL) resulted in a concentration-dependent suppression of miR-29b-3p expression (Figure 3B). Transfection of MG63 cells with a miR-29b-3p mimic significantly reduced NGF-induced PDGF-C mRNA expression and attenuated HUVEC tube formation (Figure 3C–E). These findings confirm that miR-29b-3p directly binds to the 3′-UTR of the PDGF-C gene, controlling PDGF-C expression and tumor angiogenesis in human OS cells.

### 3.3. The NGF-Overexpressing MG63 Cell Line (MG63/NGF) Enhances HUVEC Tube Formation

**Figure 4:**
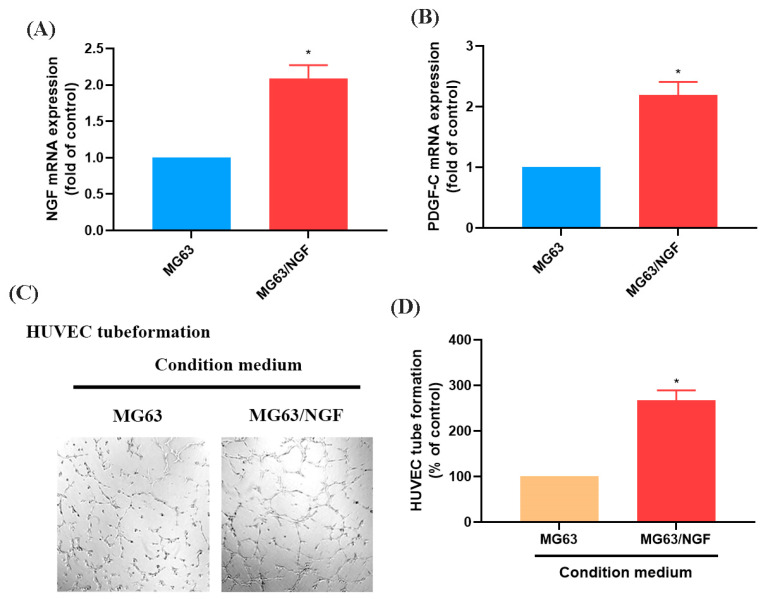
Overexpression of NGF promotes angiogenesis in MG63 cells. (**A**,**B**) MG63 cells were transfected with an empty vector or an NGF-overexpressing vector (MG63/NGF). NGF and PDGF-C mRNA expression levels were detected by qPCR. (**C**,**D**) HUVEC tube formation assays showing the growth of capillary-like structures following 6-h incubation in conditioned medium collected from MG63 and MG63/NGF cells. Images were captured at 4× magnification. All experiments were repeated at least three times. * *p* < 0.05 compared with the MG63 group.

To further investigate the role of endogenous NGF in OS, we established an NGF-overexpressing OS cell line (MG63/NGF) and confirmed increased mRNA expression via RT-qPCR (Figure 4A,B). HUVEC tube formation assays showed that MG63/NGF cells exhibited significantly enhanced angiogenic ability compared with MG63 cells, indicating that endogenous NGF strongly regulates OS angiogenesis (Figure 4C,D).

### 3.4. Larotrectinib Inhibits NGF-Induced Angiogenesis in MG63 Cells

**Figure 5:**
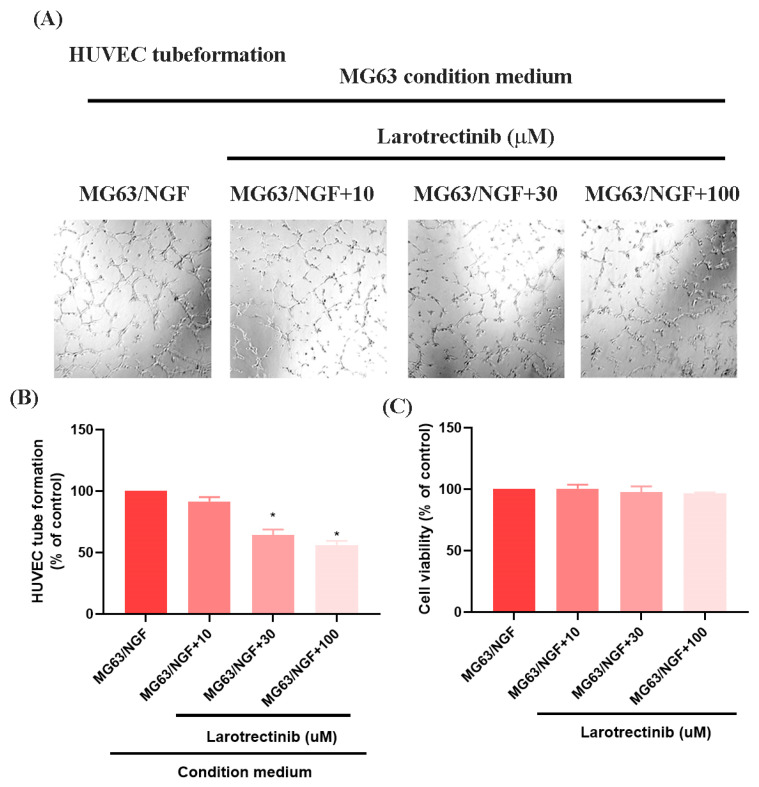
Larotrectinib suppresses NGF-induced angiogenesis in MG63 cells. (**A**,**B**) MG63/NGF cells were treated with Larotrectinib for 30 min, followed by NGF stimulation. HUVEC tube formation assays showing the growth of capillary-like structures following 6-h incubation in conditioned medium collected under these conditions. Images were captured at 4× magnification. (**C**) MG63/NGF cells were incubated with different concentrations of Larotrectinib (10, 30, 100 μM) for 24 h, and cell viability was measured using the MTT assay. All experiments were repeated at least three times. * *p* < 0.05 compared with the MG63 group.

Larotrectinib, an orally administered ATP-competitive inhibitor of the TRK family (TRKA, TRKB, and TRKC), blocks NGF signaling [35]. In HUVEC tube formation assays, Larotrectinib significantly inhibited the angiogenic ability of MG63/NGF cells, with stronger inhibitory effects observed at higher concentrations (Figure 5A,B). MTT assays revealed no significant cytotoxic effects of Larotrectinib on MG63/NGF cells at concentrations of 10, 30, and 100 μM after 24 and 48 h (Figure 5C). These findings confirm that endogenous NGF significantly enhances the angiogenic ability of MG63 cells, while Larotrectinib effectively suppresses NGF-induced tumor angiogenesis, offering a promising therapeutic strategy.

## 4. Discussion

OS is a highly vascularized malignancy where angiogenesis plays a pivotal role in tumor progression and metastasis. Angiogenesis involves the formation of new blood vessels that supply oxygen and nutrients essential for tumor growth. Recent advances in anti-angiogenic therapies targeting growth factors, chemokines, and adhesion molecules have shown promising results [36,37]. Receptor tyrosine kinases (RTKs), including EGFR, VEGFR, PDGFR, and FGFR, regulate key processes such as proliferation, angiogenesis, and metastasis in cancers like non-small cell lung cancer (NSCLC), colorectal cancer (CRC), and breast cancer [38]. Among the PDGF family, PDGF-C is a known pro-angiogenic factor, playing critical roles in OS tumor development and metastasis [39,40]. Studies have demonstrated that PDGF-C promotes endothelial cell proliferation and migration, contributing to angiogenesis in OS tumors [39]. Our study showed that NGF stimulation increased PDGF-C expression in MG63 cells, facilitating HUVEC tube formation and enhancing angiogenesis. The suppression of miR-29b-3p by NGF mediates these effects, as overexpression of PDGF-C degrades the extracellular matrix, promoting vascular remodeling. The dose-dependent effects of NGF observed in Figure 2B,E suggest that NGF plays a critical role in modulating angiogenesis through its regulation of PDGF-C expression. The dose-dependent upregulation of PDGF-C mRNA expression further underscores its pivotal role as a mediator of NGF-induced angiogenesis. This observation highlights the importance of NGF-TrkA signaling in regulating PDGF-C transcription, potentially through the suppression of miR-29b-3p, as demonstrated in this study. These findings suggest that the levels of NGF in the tumor microenvironment could influence the extent of angiogenesis and tumor progression, providing a mechanistic basis for targeting NGF-TrkA-PDGF-C signaling as a therapeutic strategy. Further investigation is warranted to explore its potential in combined or additive therapeutic approaches.

MicroRNAs are critical regulators of gene expression and have garnered attention for their roles in tumorigenesis, including OS [41,42]. Among these, miR-29b has emerged as a key regulator of OS progression. Previous studies have shown that miR-29b suppresses proliferation and migration while enhancing chemosensitivity and radiosensitivity in OS cells by targeting various oncogenic pathways, including those involved in angiogenesis [41,43]. Since angiogenesis provides the nutrients and oxygen necessary for tumor growth and metastasis, the loss of miR-29b expression creates a pro-angiogenic environment in OS tissues [43]. In addition, many studies have highlighted the critical role of miR-29b-3p in regulating angiogenesis and tumor progression across various cancer types. For instance, miR-29b-3p has been shown to negatively regulate angiogenesis by targeting VEGFA and PDGFB, thereby inhibiting endothelial cell proliferation and angiogenic activity [44]. YB-1 promotes angiogenesis in bladder cancer (BC) by downregulating miR-29b-3p, thereby upregulating VEGFA expression [45]. Additionally, methylation of the miR-29b-3p promoter in pancreatic cancer has been found to enhance angiogenesis, invasion, and migration by suppressing miR-29b-3p expression [46]. These findings collectively emphasize the pivotal role of miR-29b-3p in tumor suppression and its potential as a therapeutic target. Our study is the first to demonstrate that the NGF-TrkA axis enhances PDGF-C-mediated angiogenesis in OS by downregulating miR-29b-3p expression. This regulatory mechanism suggests that reduced miR-29b-3p levels potentiate pro-angiogenic signaling, thereby accelerating tumor progression. Restoring miR-29b-3p activity or blocking the NGF-TrkA-PDGF-C axis may offer promising therapeutic strategies. The potential use of larotrectinib, an inhibitor targeting upstream signaling components, could enhance the efficacy of such interventions. While these findings provide new insights into the molecular interplay between miR-29b-3p and angiogenesis in OS, further research is necessary to clarify the transcriptional and post-transcriptional regulation of miR-29b-3p to fully harness its therapeutic potential.

The TRK family, comprising TrkA, TrkB, and TrkC, encoded by NTRK1, NTRK2, and NTRK3, respectively, has emerged as a critical therapeutic target in cancer [47]. Neurotrophins, such as NGF, activate TRK receptors by binding to their extracellular domains, leading to dimerization and phosphorylation. This activation triggers downstream signaling pathways, including RAS/MAPKs, PI3K/AKT, and PLCγ, which regulate key cellular processes like proliferation, differentiation, and apoptosis [48]. NTRK gene fusions, identified in various pediatric and adult cancers, confer dependency on TRK tyrosine kinase activity, making TRK inhibitors promising therapeutic agents [49]. Larotrectinib, a first-generation TRK inhibitor, is an ATP-competitive molecule that has demonstrated efficacy in multiple cancers, including infantile fibrosarcoma, thyroid carcinoma, and NSCLC [5]. In our study, larotrectinib significantly inhibited NGF-induced PDGF-C expression and angiogenesis in MG63 osteosarcoma cells. By targeting the NGF-TrkA axis, larotrectinib disrupts angiogenesis-related signaling cascades, highlighting its potential as a therapeutic strategy in OS. These findings emphasize the need for further research to evaluate larotrectinib’s clinical utility in OS and other angiogenesis-dependent malignancies.

## 5. Conclusions

**Figure 6:**
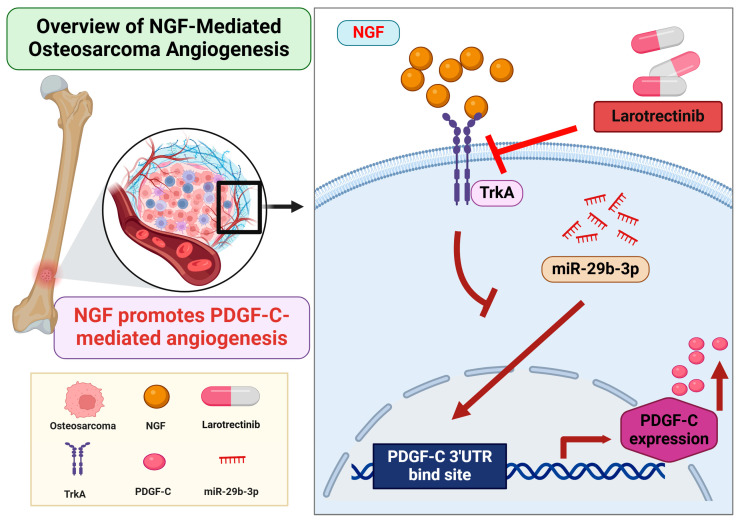
Schematic illustration of NGF involvement in OS angiogenesis. NGF facilitates PDGF-C-induced angiogenesis by suppressing miR-29b-3p synthesis in OS cells.

In summary, this study demonstrates that NGF suppresses miR-29b-3p synthesis, thereby promoting PDGF-C-dependent angiogenesis in OS cells. Larotrectinib effectively inhibits NGF-induced tumor angiogenesis, showcasing its potential as a tumor-targeted therapeutic agent (Figure 6). These findings provide valuable insights into the molecular mechanisms driving osteosarcoma pathogenesis and highlight promising avenues for the development of novel anti-angiogenic therapies. However, the findings are based on in vitro experiments, which may not fully reflect in vivo conditions. The absence of in vivo validation limits the translational potential. Additionally, other pathways involved in OS angiogenesis were not explored, and the long-term efficacy and safety of larotrectinib remain uncertain. Further in vivo studies and clinical trials are needed to confirm these findings and evaluate their clinical relevance.

## Figures and Tables

**Table 1:** List of PCR primers used for the experiments.

Target mRNA	Forward Primer (5′→3′)	Reverse Primer (5′→3′)
VEGF-A	AGGGCAGAATCATCACGAAGT	AGGGTCTCGATTGGATGGCA
PIGF	TGACATGGTTGTGCATCTGTT	ACTCTATCAGTGGTGCTCCATAC
PDGF-A	GCAAGACCAGGACGGTCATTT	GGCACTTGACACTGCTCGT
PDGF-C	ATTTGGGCTTGAAGACCCAGA	CCAGCGCCCTAATATAGTTCCA
PDGF-D	ACGGATACAGCTAGTGTTTGACA	GTCCACACCATCGTCCTCTAATA
GAPDH	ACCACAGTCCATGCCATCAC	TCCACCACCCTGTTGCTGTA
**Target miRNA**	**Forward Primer (5′** **→** **3′)**	
has-miR-29b-3p	TAGCACCATTTGAAATCAGTGTT	

## Data Availability

The RNA sequencing datasets used in this study are available in the NCBI Gene Expression Omnibus (GEO) database under accession number (GEO: GSE16088). The data generated in this study are available upon reasonable request from the corresponding author.
